# Invasion and Colonisation of a Tropical Stream by an Exotic Loricariid Fish: Indices of Gradual Displacement of the Native Common Pleco (*Hypostomus punctatus*) by the Red Fin Dwarf Pleco (*Parotocinclus maculicauda*) over Fifteen Years

**DOI:** 10.1371/journal.pone.0139968

**Published:** 2015-10-06

**Authors:** Rosana Mazzoni, Raquel Costa da Silva, Míriam Plaza Pinto

**Affiliations:** 1 Universidade do Estado do Rio de Janeiro-UERJ, Laboratório de Ecologia de Peixes-IBRAG; Rua São Francisco Xavier 524, Maracanã, Rio de Janeiro CEP 22550-013, Brasil; 2 Programa de Pós-graduação em Ecologia e Evolução/IBRAG/UERJ, Rio de Janeiro, Brasil; 3 Departamento de Ecologia, Universidade Federal do Rio Grande do Norte; Campus Universitário, Lagoa Nova, Natal CEP 59072-970, Rio Grande do Norte, Brasil; Queensland University of Technology, AUSTRALIA

## Abstract

The introduction of invasive species represents a major threat to the integrity of stream-dwelling fish populations worldwide, and this issue is receiving increasing attention from scientists, in particular because of potential impact on biodiversity. In this study, we analysed the dispersal of an exotic loricariid fish the red fin dwarf pleco (*Parotocinclus maculicauda*) in a stream of the Atlantic Forest biome in coastal south-eastern Brazil and evaluated the effects of this invasion on the native loricariid common pleco (*Hypostomus punctatus*). Specimens were collected at eight sites located along the course of the stream over a 15-year period. The distribution and density of the two species were determined by the Successive Removal Method. The introduction of *P*. *maculicauda* occurred in the medium sector of the stream, and during the course of the study, the species dispersed to new sites further upstream. By the end of the study, it was found at all points upstream from the original site. *Hypostomus punctatus* was registered at all sample sites both before and after the introduction of *P*. *maculicauda*, but its density decreased at all upstream sites after the arrival of the exotic species. Our analysis shows that colonisation by *P*. *maculicauda* seems to have a negative effect on *H*. *punctatus* densities. The maintenance of *H*. *punctatus* densities at the sites not colonised by *P*. *maculicauda* reinforces the conclusion that the colonisation of the stream by the exotic species had deleterious effects on the density of the resident *H*. *punctatus* populations, either by direct or indirect action.

## Introduction

Biological invasions are a major threat to global biodiversity and often affect ecosystem structure and function [1]. The introduction of exotic stream-dwelling fishes to local basins or wider areas is mainly the result of human activities worldwide [2]. These invasions, whether deliberate or accidental, can result in marked changes in the organisation of natural ecosystems, which can have major economic and ecological impacts [3–5], in part by altering the composition of local communities and disrupting ecosystem services [6].

Dispersal and colonisation are fundamental ecological processes that determine the distribution of populations, and their genetic structure, dynamics and persistence [7], and are fundamental components of the colonisation process. The mobility of a species and its adaptability to new environmental conditions determine the dispersal and colonisation of new habitats by exotic species [8–10]. Exotic species are considered invasive when they settle in a new area, proliferate and persist [11], threatening ecosystems, habitats and/or other species [12]. In the specific case of freshwater fishes, the success of introduction is normally very high [13], although it is important to note that not all introduced species become established [14].

Species invasions are considered one of the principal problems for the conservation of freshwater fish [15], given their potential for the reduction or even extinction of native species [16–19]. The primary route for the introduction of exotic fish species is fish farming [13], but another important source of species introductions is the release of non-native species in rivers and streams by aquarium hobbyists [11, 20] and ballast waters in coastal areas [21]. Problems resulting from this type of introduction are increasing worldwide [22].

In the vast Neotropical region, which has the highest fish diversity found anywhere in the world, exotic species have been introduced to increase the supply of protein for local human populations (e.g., *Tilapia niloticus*) or to increase the productivity of new reservoirs, as in the case of the Amazonian corvine, *Plagioscion squamosissimus*, which was translocated to many reservoirs in the Paraná basin of southern Brazil. The peacock bass (*Cichla ocellaris*) has also been introduced into many areas for the purposes of sport angling, and there have been numerous accidental introductions from fish farms. A more recent phenomenon, as mentioned above, is the introduction of species by aquarium enthusiasts. Despite the widespread nature of these processes, very little is known of their impacts on natural populations and their habitats. There are much more data about the implications of invasion by exotic species from the temperate northern hemisphere where, for example, [23] documented the complete replacement of the brook trout by the brown trout in a Minnesota stream over a 15-year period, a process supported by the innate behaviour of the species, together with local habitat disturbance.

In the present study we describe the replacement of a native illiophagous loricariid fish in south-eastern Brazil by a morphologically and ecologically similar species introduced recently into the stream, probably by aquarium enthusiasts. We analysed the dispersal of the exotic red fin dwarf pleco, *Parotocinclus maculicauda* (Steindachner, 1877) in the stream, and evaluated how its density varied over time and in space, and how this affected the density of the native common pleco, *Hypostomus punctatus* Vallenciennes, 1840. These closely-related loricariids inhabit the benthic zone of streams, where they forage and reproduce [24]. Given their phylogenetic and ecological similarities, we hypothesised that the introduction of the invasive species was likely to have a negative effect on the resident, native species.

## Methods

### Ethics Statement

This study was carried out in strict accordance with the recommendations contained in the Guide for the Ethics Committee for the Care and Use of Experimental Animals (Comissão de Ética para o Cuidado e Uso de Animais Experimentais—CEUA/Brazil) of the Brazilian College of Animal Experimentation (Colégio Brasileiro de Experimentação Animal—COBEA/Brazil). The protocol was approved by CEUA/Brazil under permit number 012/2013 (April 2nd 2013). Field authorization was provided by the Brazilian agency IBAMA—authorization Sisbio number 1.916.854.

### Study Area

The present study was conducted in the Rio Ubatiba, a typical small watercourse of the lowland Serra do Mar region, 70 km north of Rio de Janeiro (Brazil). This stream is approximately 16 km long, and drains a basin of 42 km² at altitudes of below 30 m above sea level. Most of this basin coincides with land deforested for plantations and cattle ranching, although some patches of pristine Atlantic Forest persist on the rocky slopes and peaks of some of the surrounding hills.

The Ubatiba discharges into Maricá Lagoon, a large, brackish water lagoon formed by the accumulation of offshore sand dunes. Water flow is regulated solely by rainfall (~1500 mm.y^−1^) with a maximum run-off between October and January (~1300 mm) and minimum between May and July (~150 mm). Unpredictable tropical storms (with precipitation of more than120 mm in a single day) may nevertheless occur throughout the year, resulting in a three-fold increase in water discharge within a few hours. The fish fauna of the Rio Ubatiba includes a total of 22 species [25].

### Sampling and Data Analysis

**Fig 1:**
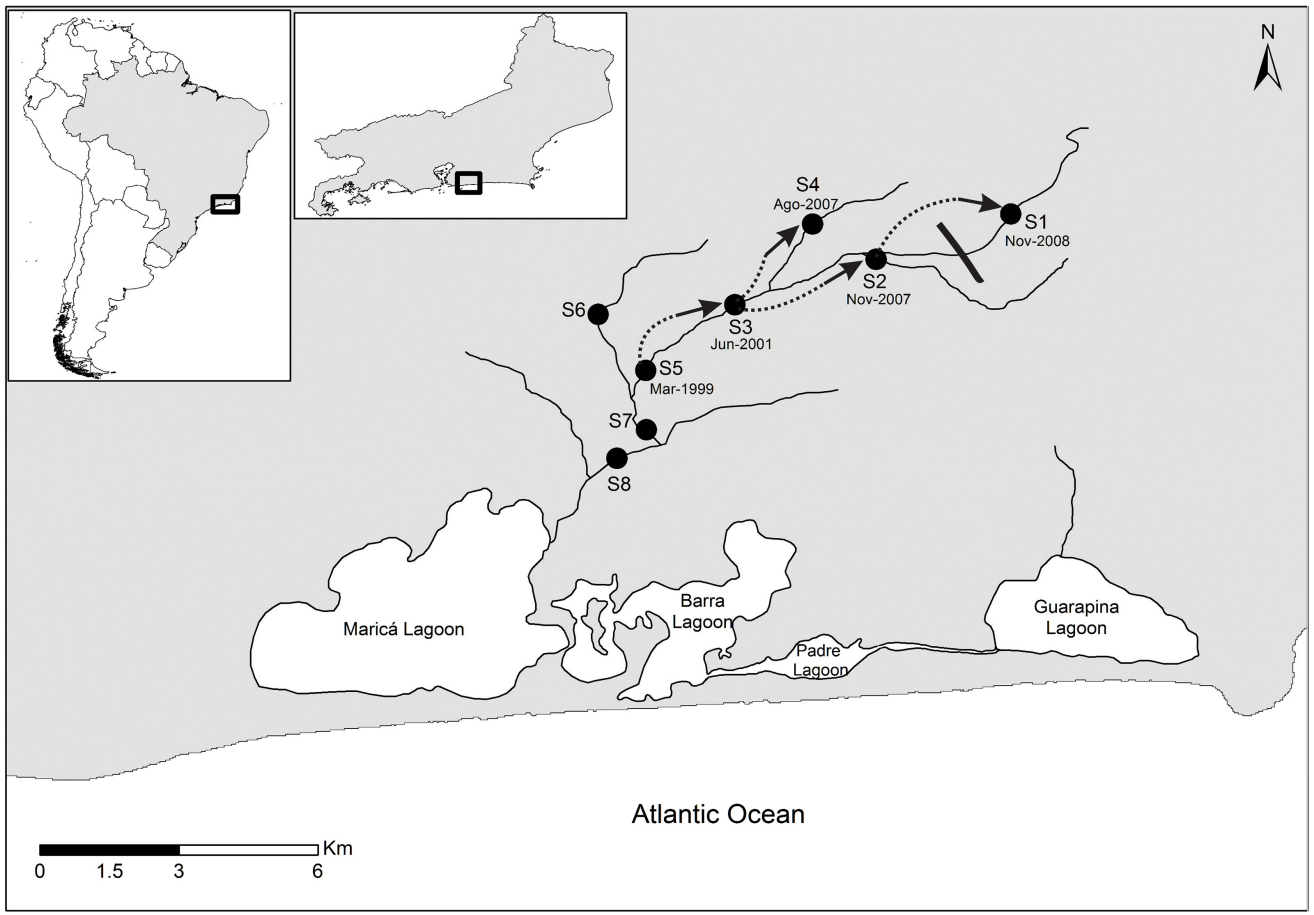
Study area and the dispersal of *P*. *maculicauda*. Fig 1. Geographic location of Ubatiba stream system showing the dispersal of the dwarf pleco *Parotocinclus maculicauda* until 2009, following its introduction in 1999. The arrows indicate its suggestive dispersal, and the dates refer to the first record of the species at the respective site. The black line between sites 1 and 2 indicates the location of a waterfall.

Samples were collected at the following eight sites (Fig 1) located along the course of the stream: site 1 (22°52.30’S, 42°44.23’W), site 2 (22°52.43’S, 42°44.46’W), site 3 (22°51.93’S, 42°44.90’W), site 4 (22°51.67’S, 42°44.52’W), site 5 (22°51.93’S, 42°44.52’W), site 6 (22°52.27’S, 42°47.01’W), site 7 (22°53.60’S, 42°47.46’W) and site 8 (22°53.60’S e 42°47.46’W). The samples taken in this study followed the sampling design proposed for a long-term project on the stability of the stream-dwelling fish community from Ubatiba stream. All sites were always visited at each sampling occasion. These sites (100 m long) were selected to include the variability of each particular stream reach. We conducted quantitative sampling every second month from July 1994 to September 2009 (details in Table 1). Samples were obtained by electrofishing (900 W, 2–3 A) through the three-removal method. At the end of the last removal, each captured fish was measured (standard length, *SL*, cm) and returned alive to the water. Fish abundance was estimated by applying the 3- removal method [26] and transformed into densities (ind.ha^−1^) based on the sampled area of each site. Sampled area was calculated for each sampling month and site, according to bathymetric maps based on transversal transects registered within an interval of 5 m long, from the lower to the upper section of the sampling site. All fish collected were measured (mm) and released back to the water at a mid point of the sampling site. Capture data for single species and dates were tested for homogeneity of catchability (p) between successive removals and for failure condition [26, 27]. For details on the sampling design, regularity, and the electrofishing characteristics, please see [25 and 28].

**Table 1:** Frequency of electrofishing samples collection at the Rio Ubatiba between 1994 and 2009.

Sample frequency	Period
Bimonthly	July 1994 to August 1997
Monthly	February to March 1998
Bimonthly	March to July 1999
Bimonthly	April to November 2000
Bimonthly	April to August 2001
Bimonthly	July 2007 to May 2008
Monthly	November 2008
Bimonthly	July to September 2009

To characterize the colonisation process at each study site, we conducted a spatiotemporal body-size (standard length) distribution analysis for *P*. *maculicauda*. Populations of this species were considered to have been successfully established at a given site when a shift in the histogram to higher frequencies of the smaller body-size classes was observed. Standard length at first maturity in *P*. *maculicauda* is 3.0 cm (unpublished data).

We used a Spearman correlation and model selection with a multiple linear regression approach [29] to investigate whether *H*. *punctatus* densities were related to those of *P*. *maculicauda* and/or environmental factors. Variation in these environmental factors was summarised using a Principal Components Analysis (PCA, [30]). The environmental variables analysed were (i) stream substratum, (ii) volume of rainfall, (iii) canopy and in-stream vegetation, (iv) stream width and depth, and (v) conductivity.

We evaluated the relationship between the density of *H*. *punctatus* (response variable) and *P*. *maculicauda* with the first PCA axis, and the second PCA axis (predictor variables) using a linear regression with a model selection approach. We also evaluated this relationship using a nonparametric Spearman correlation because the dependent variable did not have a normal distribution and the residuals violated the assumption of homogeneity of variances. All analyses were performed in Statistica 11 and R [31].

## Results

**Fig 2:**
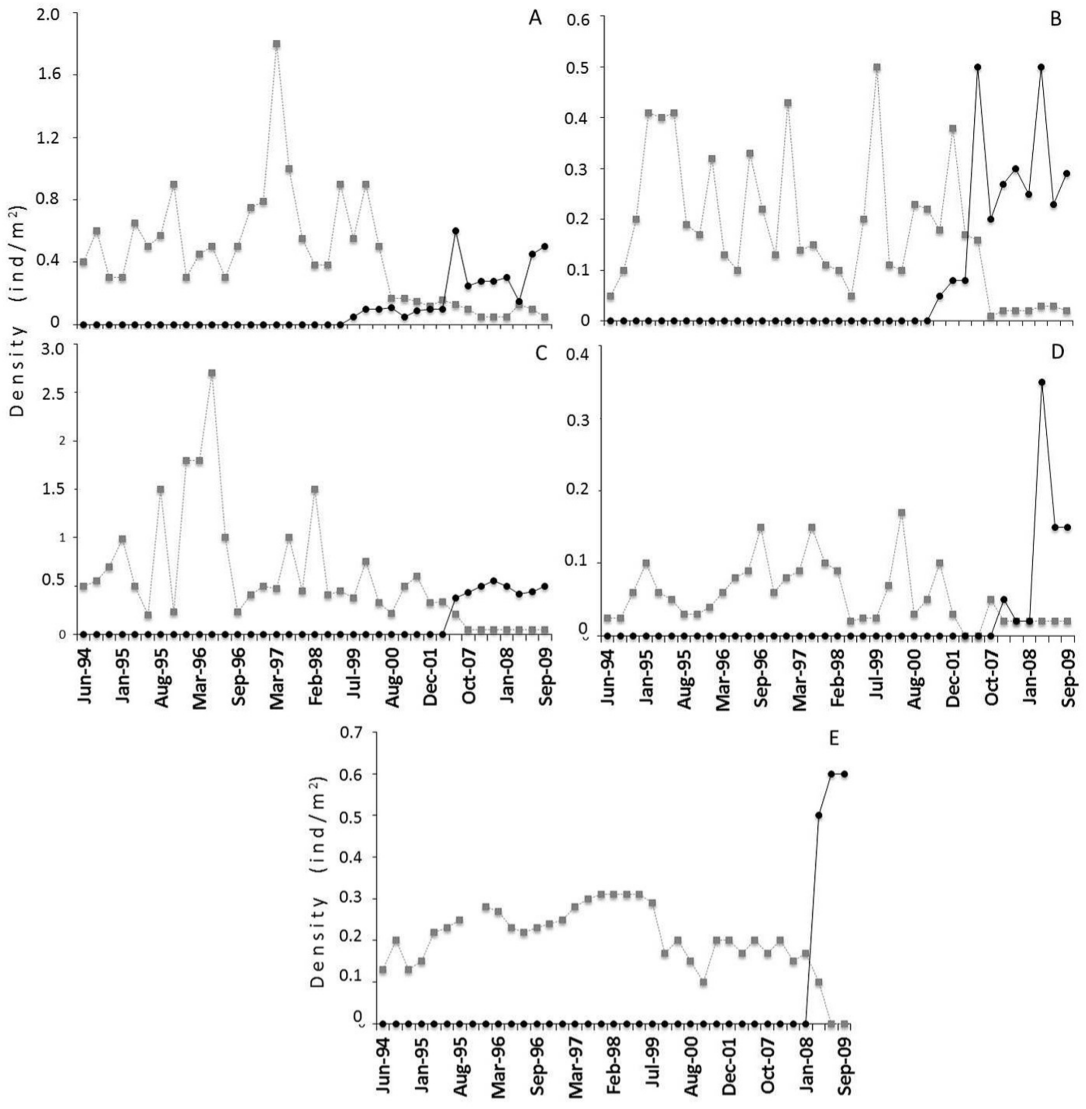
Upstream densities of *Parotocinclus maculicauda* and *Hypostomus punctatus*. Fig 2. Temporal variation in the density of *Parotocinclus maculicauda* (black circles) and *Hypostomus punctatus* (grey squares) in the Rio Ubatiba at sites (A) 5, (B) 3, (C) 4, (D) 2, and (E) 1. Note that Y-axis is not at the same scale.

Between July 1994 and June 1999, no *P*. *maculicauda* were captured in the Ubatiba stream. The first record of *P*. *maculicauda* was obtained in March, 1999, at site 5, when an extremely low density (0.05 individuals/m²) was registered (Fig 2A). As the study continued, *P*. *maculicauda* was registered in areas progressively further upstream. In June 2001, the species was recorded for the first time at site 3 at a density of 0.05 ind/m² (Fig 2B), and in August 2007, specimens were caught for the first time at site 4, with a density of 0.38 ind/m² (Fig 2C). In November 2007, *P*. *maculicauda* was recorded for the first time at site 2, with a density of 0.05 ind/m² (Fig 2D), and a year later, the same density was recorded at site 1 (Fig 2E). It is interesting to that the latter site (1) is located just upstream from a waterfall of approximately 4 m in height.

**Fig 3:**
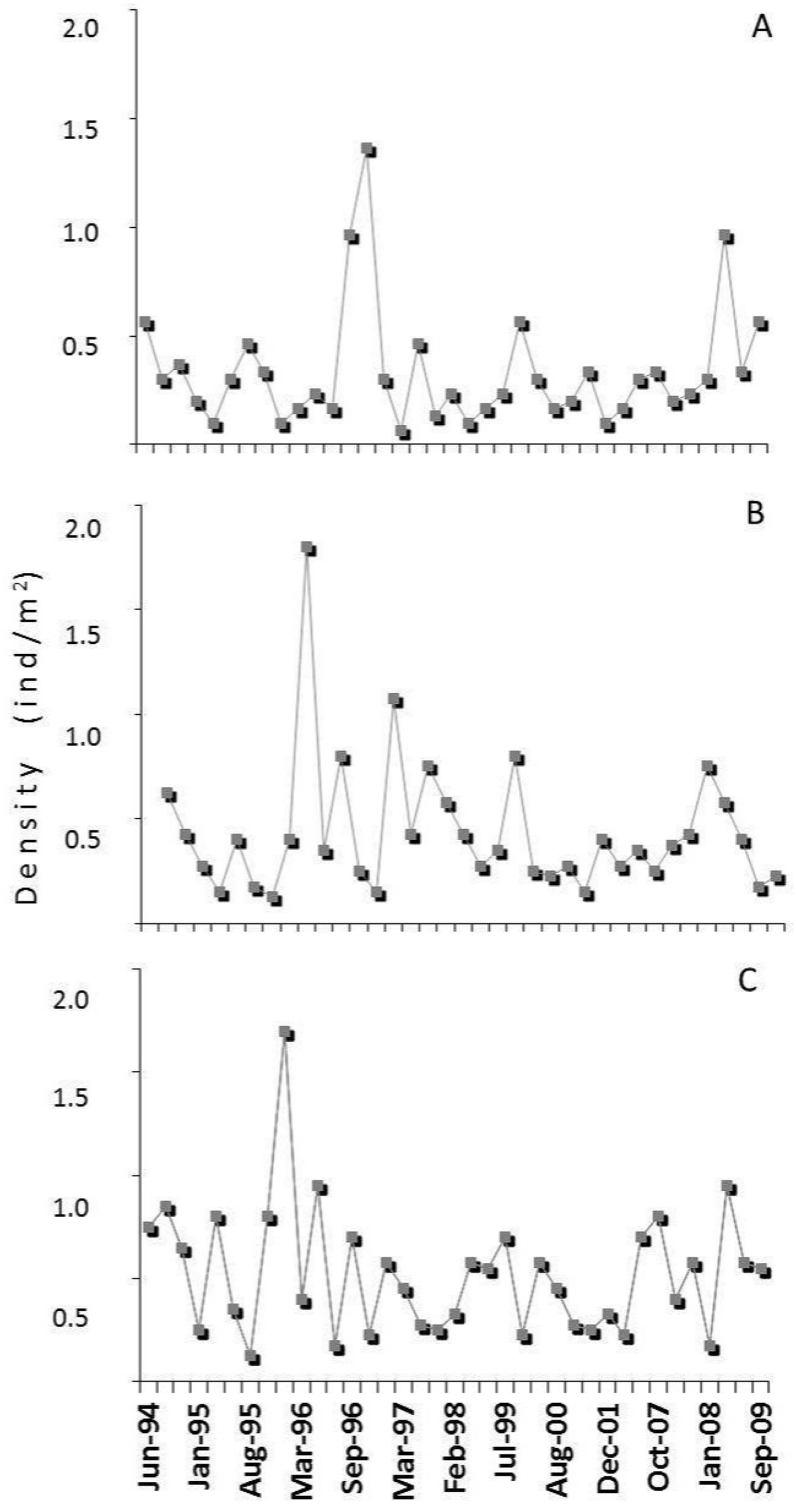
Downstream densities of *Hypostomus punctatus*. Fig 3. Temporal variation in the density of *Hypostomus punctatus* in the sites of Rio Ubatiba sites (A) 6, (B) 7, and (C) 8.

By the end of the study period, *P*. *maculicauda* was found at all sites upstream from site 5, where it was recorded for the first time. During the same period (1994–2009), samples from sites 6 to 8, which were downstream from site 5 and were not invaded by *P*. *maculicauda*, indicated that the density of *H*. *punctatus* remained constant over the study period (Fig 3).

The native *H*. *punctatus* was found at all sites both before and after the introduction of the exotic *P*. *maculicauda*. However, the density of *H*. *punctatus* decreased at all sites (1–5) once *P*. *maculicauda* arrived (Figs 1A, 1B, 1C, 1D and 1E). We recorded negative correlations between the density of *P*. *maculicauda* and that of *H*. *punctatus* for the entire study period after the introduction of *P*. *maculicauda* (Spearman r = -0.38, p < 0.01, n = 159) and for the period after 1999 (r = -0.41, p < 0.01, n = 80).

**Fig 4:**
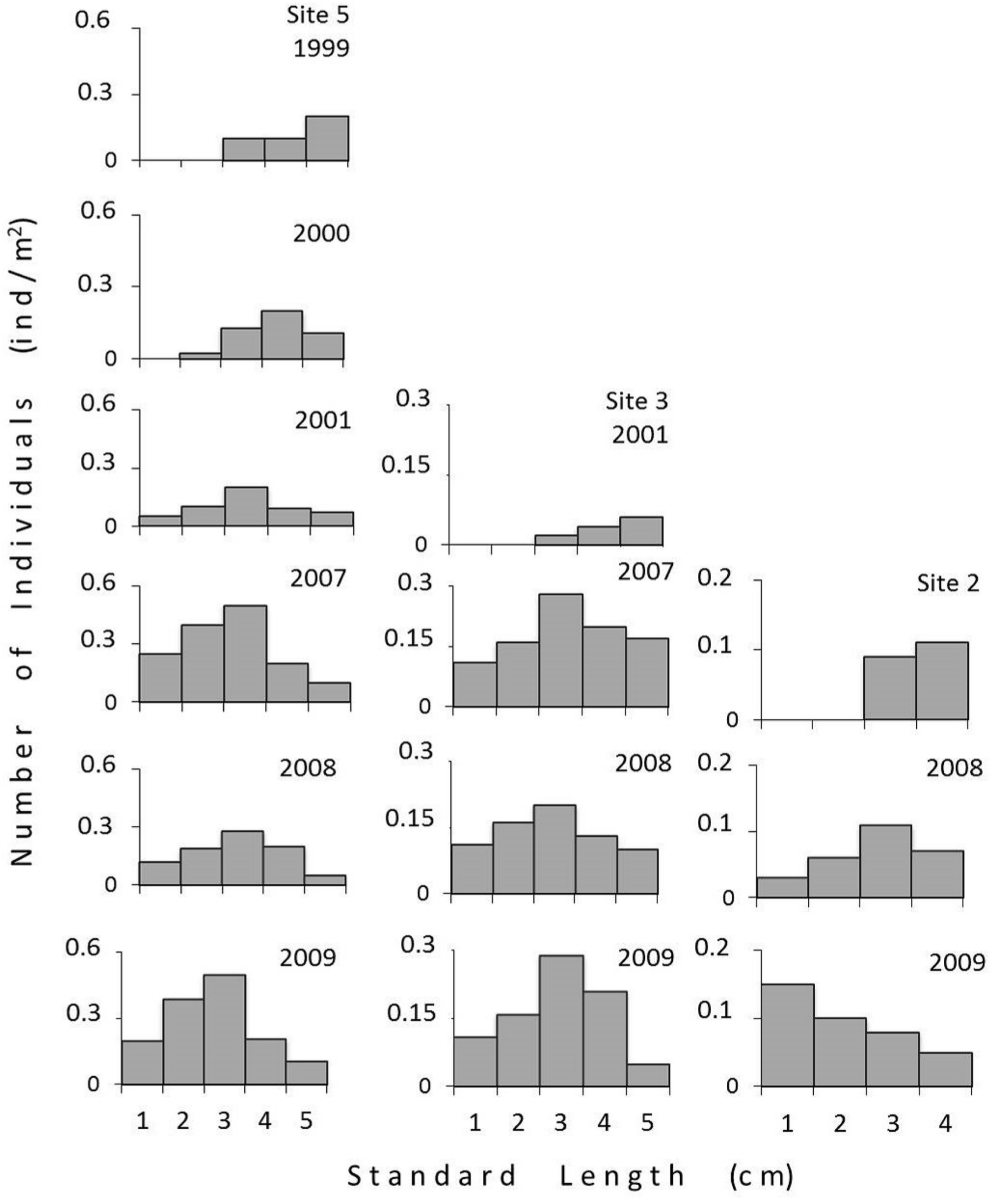
Body size distribution of *Parotocinclus maculicauda*. Fig 4. Distribution of *Parotocinclus maculicauda* body size classes at sites 5, 3, and 2 between 1999 and 2009.

There were some shifts in the body-size distribution of *P*. *maculicauda* (Fig 4). Site 5 was the first to be invaded in 1999, when we recorded only adult individuals, with body length of over 3 cm. In 2000, the first juveniles (body length < 3.0 cm) were recorded, and a well-structured population with individuals of several different body-size classes was observed in 2001 and subsequent years (2001, 2007, 2008, 2009).

The invader species was first recorded at site 3 in 2001, but once again, only adults were observed initially, and colonisation was only confirmed in 2007, when all size classes, including juveniles and adults, were observed simultaneously. It is important to note, however, that there was a gap in sampling between 2001 and 2007, which means that the colonisation of this site occurred at any time between in the six years following the first appearance of the invader species. A similar pattern was observed at site 2 (Fig 4).

The first and second PCA axes explained 45% and 22% of the environmental variation, respectively (S1 File). The model that best explained the variation in the density of *H*. *punctatus* included *P*. *maculicauda* densities and the second PCA axis (Akaike weight = 0.68, see Table 2). This was also confirmed by the Spearman correlation (Table 3). The density of *H*. *punctatus* density was related negatively to that of *P*. *maculicauda* (r = -0.41; p < 0.01) and to the second PCA axis (r = -0.43; p < 0.01).

**Table 2:** The eight different models based on the density of *Hypostomus punctatus* at the five sampling sites as the response variable, with all the possible combinations of independent variables (density of *Parotocinclus maculicauda*, and first and second PCA axes) were fitted and compared using Akaike’s Information Criterion and the relative weight (*w*_i_) for each model. The best model, with the highest *w*_*i*_ value, includes the predictors *P*. *maculicauda* density and the second PCA axis.

Model	Independent variable (coefficient)	*R* ^2^	S	Delta AIC	*w*_*i*_
**1**	Intercept only		-78.6	18.07	< 0.001
**2**	Density of *P*. *maculicauda* (-0.238)	0.09	-84.5	12.24	0.002
**3**	First PCA axis (-0.006)	0.01	-77.4	19.27	< 0.001
**4**	Density of *P*. *maculicauda* (-0.259) First PCA axis (-0.004)	0.10	-82.6	14.08	0.001
**5**	Second PCA axis (-0.029)	0.14	-88.7	8.04	0.012
**6**	Density of *P*. *maculicauda* (-0.238) Second PCA axis (-0.030)	0.24	-96.7	0.00	0.685
**7**	First PCA axis (-0.006) Second PCA axis (-0.030)	0.15	-87.6	9.14	0.007
**8**	Density of *P*. *maculicauda* (-0.272) First PCA axis (-0.005) Second PCA axis (-0.030)	0.25	-95.9	1.70	0.293

**Table 3:** Pairwise Spearman correlations between the densities of *H*. *punctatus* and *P*. *maculicauda*, and the first and second PCA axes. The correlation coefficient, *r*, is shown above the diagonal, and tCe *P* values are shown below the diagonal (italics).

	Density of *H*. *punctatus*	Density of *P*.*maculicauda*	First PCA axis	Second PCA axis
**Density of *H*. *punctatus***	-	-0.41	0.15	-0.43
**Density of *P*. *maculicauda***	*< 0*.*01*	-	-0.66	-0.05
**First PCA axis**	*0*.*18*	*< 0*.*01*	-	0.10
**Second PCA axis**	*< 0*.*01*	*0*.*68*	*0*.*40*	-

## Discussion

The dispersal route of the invasive red fin dwarf pleco (*P*. *maculicauda*) can be inferred from the samples collected between July 1994 and September 2009. After being recorded for the first time at a site in the medium sector of the stream (site 5), *P*. *maculicauda* dispersed upstream to colonise new sites. By the end of the study, the species was found at five sites, indicating a high dispersal capacity, an important ability for an invasive species [32].

A number of studies have attributed the ability of certain fish species to recolonise a community following a drought to their dispersal capacity [33, 34], although other studies have argued that this capacity is not the primary factor determining successful colonisation [35–37]. Colonisation may be controlled by a variety of distinct factors on different spatial scales [37]. The abundance of the species, its density, the distance from its sources, and fluctuations in environmental variables and habitat structure are among the principal factors that determine the rate and success of colonisation by a fish species [9, 33, 34, 36, 37]. Unfortunately, the lack of data on the dispersal patterns of most Neotropical species hampers the systematic understanding of the relationship between dispersal and colonisation [38].

In the Rio Ubatiba, *P*. *maculicauda* was able to disperse successfully throughout much of the watercourse [39]. The dispersal capacity of this species and its ability to withstand the variation in physical, chemical, and ecological characters would have underpinned its ability to colonise the stream. Its dispersal routes may also have been influenced by the morphology of the species and anthropogenic changes in the environment. Understanding these dispersal routes is important for the definition of the factors that limit the distribution of a species and other aspects of its ecology, and provides important insights for the development of management and conservation programs [7].

During the study period, *P*. *maculicauda* was not only able to disperse to all the sites upstream from site 5, where it was found originally, but also established populations at all locations. The same pattern of colonisation was observed at all sites, with only larger individuals (adults) being found in the first samples, followed by a shift in the body-size distribution over time, with an increasing contribution of smaller individuals. This indicates that the dispersing adults were reproducing and establishing permanent populations at the new sites. Beyond the evidence, other hypothesis can also explain the shifts in the age-structure observed, such as an increased mortality of adults or dispersal events, either emigrations of adults or immigrations of juveniles.

The density of *P*. *maculicauda* also shifted from very low initial levels to values similar to those of the original *H*. *punctatus* populations (Fig 1). Exotic species may compete with native ones or suppress their populations [32, 40]. In the present study, the high densities recorded for the introduced species and the decline in the density of the native species indicate that the introduction of *P*. *maculicauda* had a negative effect on the resident population of *H*. *punctatus*. This was corroborated by the fact that the model which best explained the *H*. *punctatus* densities included the second PCA axis (which represented the environmental characteristics of the sample sites) and *P*. *maculicauda* densities. The Spearman correlation returned similar results. Furthermore, *P*. *maculicauda* was represented by reproductively active individuals (unpublished data) at all sites on the Rio Ubatiba, supporting viable local populations. These findings support the classification of *P*. *maculicauda* as an exotic invasive species.

In 1999, the ornamental species *P*. *maculicauda* was introduced into the Rio Ubatiba, probably by aquarium hobbyists. Ornamental fish, which are commonly kept in aquaria for educational, aesthetic or entertainment purposes, are among the most frequently introduced vertebrate groups worldwide [41]. Reproductive rates, territoriality, parental care, resistance to desiccation, and the ability to use atmospheric oxygen are among the features that facilitate the colonisation of new habitats by loricariid catfishes, in comparison with most other species groups [42]. Worldwide, exotic locariids introduced into novel habitats have successfully colonised almost 80% of the sites [43].

The native species, *H*. *punctatus*, was found at all sites along the Rio Ubatiba prior to the introduction of the exotic *P*. *maculicauda*. [44] also recorded *H*. *punctatus* in the Ubatiba between June 1987 and July 1988. The native species persisted at all sites by the end of the study, although its density was greatly reduced at the sites colonised by *P*. *maculicauda* (Fig 1). While *H*. *punctatus* did not suffer local extinctions, there is a possibility that *P*. *maculicauda* caused a decline in *H*. *punctatus* densities.

Although we have evidence of the exotic invasive species effects on the native one, we should warily discuss other possible scenarios. Changes in the abundance of both species could have been caused by different environmental factors, such as climatic variations, climate change, interactions with other species. Otherwise, *H*. *punctatus* may have declined due to other factors, which made its habitat free for passive colonization by *P*. *maculicauda*.

Continued monitoring of these populations will be important to determine whether *H*. *punctatus* will be able to maintain a viable population—albeit at much lower densities than before—in the Rio Ubatiba over the long term. It would be insightful to understand other aspects of the life histories of the two species in order to better evaluate the effects of the introduction of *P*. *maculicauda* not only on the resident *H*. *punctatus* population, but also the remaining species of the local fish community. The persistence of *H*. *punctatus* densities at the sites (six and eight) not colonised by *P*. *maculicauda* is also evidence of the deleterious effects of the colonisation of the stream by *H*. *punctatus*.

## Supporting Information

S1 File Principal Component Analysis (PCA) for environmental variables. (PDF)
