# Supplementary material for: Invasion and Colonisation of a Tropical Stream by an Exotic Loricariid Fish: Indices of Gradual Displacement of the Native Common Pleco (Hypostomus punctatus) by the Red Fin Dwarf Pleco (Parotocinclus maculicauda) over Fifteen Years
Source: PLoS One. 2015 Oct 6;10(10):e0139968. doi: 10.1371/journal.pone.0139968 (PMC4595082; doi:10.1371/journal.pone.0139968)
Supplement: S1 File — (PDF) [file pone.0139968.s001.pdf]

SUPPORTING INFORMATION

Invasion and colonisation of a tropical stream by an exotic loricariid fish: gradual displacement of the native common pleco (*Hypostomus punctatus*) by the red fin dwarf pleco (*Parotocinclus maculicauda*) over a fifteen years

Rosana Mazzoni, Raquel Costa da Silva, Míriam Plaza Pinto

The environmental variation was summarized using Principal Component Analysis (PCA). The first PCA axis accounts for 45% of the data variance and the second axis accounts for 22% of the data variance. We present the PCA 1 and PCA 2 cases coordinates projected in a scatterplot (Figure S1). We also present PCA1 and PCA2 variables coordinates at Table S1, which show the relative contribution of each environmental variable to the axis.

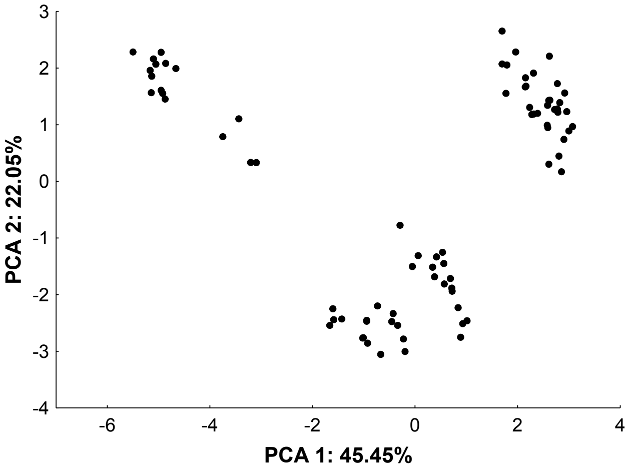

Figure S1: Projections of the cases coordinates of Principal Component Analysis 1st and 2nd axis. The environmental descriptors are: total rain, mesohabitat data (pools, riffles, runs), substratum data (mud, clay, sand, gravel, small boulder, large boulder and bedrock), canopy cover, instream vegetation, stream width, stream water column maximum depth and conductivity.

Table S1: Contributions of the different descriptors to the formation of principal axis 1 and 2. The environmental descriptors are: (1) total rain, mesohabitat data ((2) pools, (3) riffles, (4) runs), substratum data ((5) mud, (6) clay, (7) sand, (8) gravel, (9) small boulder, (10) large boulder, (11) bedrock), (12) canopy cover, (13) riverine vegetation, (14) stream width, (15) stream water column maximum depth, (16) conductivity.

Descriptor	PCA 1	PCA 2
**1**	0.104	-0.148
**2**	-0.107	0.910
**3**	0.891	0.278
**4**	-0.802	-0.486
**5**	-0.410	0.262
**6**	-0.525	0.553
**7**	-0.765	0.405
**8**	-0.288	-0.458
**9**	0.766	-0.511
**10**	0.666	0.539
**11**	-0.855	0.425
**12**	0.757	0.607
**13**	-0.770	-0.599
**14**	0.586	-0.058
**15**	-0.803	0.150
**16**	-0.887	0.360
